# Near‐Death Experience During Emergency Ketamine Use: A Case Report

**DOI:** 10.1002/brb3.70939

**Published:** 2025-10-24

**Authors:** Pauline Fritz, Anaïs Pichelin, Aurore Ancion, Naji Alnagger, Nicolas Lejeune, Alexandre Ghuysen, Olivia Gosseries, Charlotte Martial

**Affiliations:** ^1^ Coma Science Group, GIGA‐Consciousness University of Liège Liège Belgium; ^2^ NeuroRehab and Consciousness Clinic, Neurology department University Hospital of Liège Liège Belgium; ^3^ Department of Emergency University Hospital of Liège Liège Belgium

**Keywords:** emergency care, ketamine, near‐death experiences, psychological transformation

## Abstract

**Purpose:**

Survivors from acute life‐threatening conditions occasionally report various alterations in subjective experiences, ranging from visual perceptual changes to multi‐faceted phenomena that can be categorized as near‐death experiences (NDEs). We report an NDE in a 73‐year‐old woman with an acute exacerbation of chronic obstructive pulmonary disease, requiring emergency intubation and mechanical ventilation for acute respiratory failure.

**Method:**

The patient suffered from profound hypoxia and hypercapnia with uncompensated respiratory acidosis. Prehospital anesthesia was induced with an intravenous bolus of 500 mg of ketamine administered over 60 s, corresponding to 7 mg/kg of adjusted body weight. The patient eventually recovered and was weaned from ventilator after 24 h. Semi‐structured interviews, including several standardized scales assessing potential subjective experience and post‐event impact, were conducted at 2 weeks and 2 months post‐event.

**Finding:**

Findings revealed a vivid NDE (i.e., meeting the NDE Content scale criteria, with total scores of 34/80 and 32/80 at the first and second interviews, respectively), notably characterized by feelings of peacefulness and well‐being, encounters with entities, and the perception of a bright light. Over time, the narrative evolved, with some elements no longer recalled at follow‐up, while the patient reported a positive psychological transformation, including improved mood and increased empathy.

**Conclusion:**

This case, set within its medical and pharmacological context, provides a unique opportunity to study NDEs and their precipitating context. The reported transformations underscore the importance of recognizing NDEs for their possible long‐term effects on patients and relevance to medical practice.

## Graphical abstract

We report a near‐death experience in a 73‐year‐old woman with acute respiratory failure who received an accidental 500 mg ketamine dose during intubation. Semi‐structured interviews revealed a vivid and evolving NDE narrative, followed by lasting psychological transformation, underscoring the clinical and research significance of NDEs in critical care.

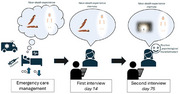

## 1 Introduction

“I arrived as if on a walkway, everything was all white. There were a lot of people, I suppose they were family members… And beyond that crowd, there was a great light that, while bright, was calming. I hope to see it again someday. […] One shouldn't fear death. […] At some point, someone—or something, I don't know—placed a hand on me. I couldn't go through. That person, that angel, stood between the people and me, I couldn't pass, it wasn't my time.*”* Translated verbatim from French, this excerpt is drawn from the freely expressed narrative provided during the first interview of this case report, conducted 2 weeks after the patient's admission to the hospital.

Emergency departments serve as the frontline for critical medical interventions, often requiring rapid and decisive actions in patient care management. In these urgent scenarios, various medications can be used to facilitate high‐risk procedures such as endotracheal intubation. Among the medications employed for such procedures, ketamine stands out for its favorable cardiorespiratory safety profile. In addition to its rapid‐onset sedative and analgesic properties, unlike other anesthetics, ketamine preserves respiratory function and cardiovascular stability. These unique properties make it particularly useful in life‐threatening situations (Kim et al. 2021; Matchett et al. 2022). Ketamine is commonly classified as a dissociative anesthetic, yet its unique phenomenological profile, which shares considerable overlap with classical psychedelics, complicates its categorization. In emergency medicine, the standard induction dose for rapid sequence intubation typically ranges from 1 to 2 mg/kg intravenously (IV). While pharmacological guidelines allow doses up to 4.5 mg/kg, 2.5 mg/kg is often considered the upper limit in acute care settings (Engstrom et al. 2023; Green et al. 2011).

Qualitative reports show that participants experience various dissociative effects under ketamine, be it from their environment, physical body, or sense of self (Marguilho et al. 2023). At sufficiently high doses, ketamine can induce what recreational users term a “k‐hole” (Curran and Monaghan 2001; Nicol and Morton 2020), in which ongoing mental activity becomes disconnected from external stimuli; in some instances, this state progresses into out‐of‐body experiences (OBEs), whereby experiencers perceive themselves as detached from their own body. Additionally, ketamine induces a range of perceptual distortions, including alterations in spatial and depth perception, as well as auditory distortions. Ketamine can also induce changes in the narrative self, which can manifest as ego dissolution, a profound sense of interconnectedness, and characteristics more commonly associated with classical psychedelics and captured by the ego dissolution scale (EDS) (Sleight et al. 2023). Interestingly, many of these phenomenological features share striking similarities with near‐death experiences (NDEs). This was shown in a study using natural processing to compare the written experiential accounts of 165 different substances (commonly referred to as trip reports) with the written narratives of classical NDEs, revealing that ketamine experiences presented the highest semantic similarity with NDE reports (Martial et al. 2019).

Classical NDEs can be defined as an episode of disconnected consciousness (i.e., internal awareness during a period of unresponsiveness) that typically occurs in critical, potentially life‐threatening conditions (Martial, Cassol, et al. 2020). These episodes are characterized by specific prototypical features, such as experiencing sensations of peacefulness/well‐being, OBEs, seeing a bright light, an altered sense of time, and entering a different, unearthly environment (Charland‐Verville et al. 2014), and can be identified using the NDE‐Content (NDE‐C) scale (Martial, Simon, et al. 2020). Prospective studies in populations experiencing life‐threatening situations (e.g., cardiac arrest, intensive care unit [ICU] stay) have suggested a prevalence of NDEs between 4% and 23% (Greyson 2003; Hou et al. 2013; Klemenc‐Ketis et al. 2010; Parnia et al. 2023, 2014; Rousseau et al. 2023; Schwaninger et al. 2002; van Lommel et al. 2001).

Interestingly, both ketamine‐induced experiences and NDEs have been described as transformative, with lasting impacts on life and attitudes toward death, including, in the case of ketamine, sustained antidepressive and anti‐addictive properties (Bahji et al. 2021; Dakwar et al. 2018; Noyes 1980; Phillips et al. 2020; Rothberg et al. 2021; Sweeney et al. 2022). In parallel, the mechanisms of NDEs have gained growing interest, notably through the recent NEPTUNE model, which encompasses various explanatory perspectives (Martial et al. 2025). These include cognitive explanations, such as a greater propensity for dissociation in NDE experiencers, as well as neurophysiological hypotheses linking intense subjective experience to physiological stress, notably altered blood gas levels like hypoxia and hypercapnia (Lempert et al. 1994; Martial, Piarulli, et al. 2024). A prospective study, for instance, showed higher concentrations of carbon dioxide in people experiencing an NDE following out‐of‐hospital cardiac arrest (Klemenc‐Ketis et al. 2010). In addition, certain neurotransmitter systems, such as the glutamatergic system, which notably includes N‐methyl‐D‐aspartate (NMDA) receptors, are thought to be involved by different mechanisms in both ketamine‐induced experiences and NDEs, which may account for their phenomenological similarity. Given the overlapping phenomenology and lasting impacts reported in both NDEs and ketamine‐induced states, one might wonder whether the presence of ketamine could precipitate an NDE that would not have occurred otherwise or intensify its content or subjective depth. Equally intriguing is how these two similar phenomena intersect and what this convergence might reveal about the underlying mechanisms of such experiences.

This case report describes a 73‐year‐old Belgian female with a non‐infectious exacerbation of chronic obstructive pulmonary disease (COPD) with acute‐on‐chronic ventilatory failure. Due to a miscommunication about drug dilution between the emergency physician and the nurse and likely facilitated by the emergency context, an IV ketamine bolus was inadvertently administered at 2.5 times the usual dose. We here report the acute care management and the results of two structured follow‐up interviews. This case report was conducted according to the guidelines of the Declaration of Helsinki, after approval by the Ethics Committee of the Medicine Faculty of the University of Liège (2022‐389) and registration on ClinicalTrials.gov (NCT06362525).

## 2 Case Report

### 2.1 Medical History and Acute Care Management

The patient's medical history included Gold IV‐COPD, class III obesity (104 kg for 155 cm), and stage IIIb chronic kidney disease with a creatinine blood level baseline at 1.31 mg/dl. She also had a history of depression but no known neurological disorders.

In the days preceding hospital admission, the patient experienced progressive worsening of her baseline dyspnea, leading to an increase in her home oxygen supply from 2 to 6 L/min. The acute event occurred when her husband called the ambulance after finding her unresponsive for several hours. At the first medical contact, she was found with an altered level of consciousness (Glasgow Coma Scale score: 3/15) with compromised airway and severe hypoxemia with hemoglobin oxygen saturation (spO_2_) measured at 50% (normal range for COPD 88%–92%), prompting immediate preparation for endotracheal intubation. The initial hemodynamic status was preserved with a heart rate at 80/min and blood pressure at 140/70 mmHg. The measured glycemia was elevated at 230 mg/dl. Induction of anesthesia was performed using an IV bolus of ketamine (500 mg, corresponding to 7 mg/kg based on an adjusted body weight calculated as Ideal Body Weight + 0.4 × [Total Body Weight—Ideal Body Weight]), in combination with a neuromuscular blocker (suxamethonium 100 mg). The intubation was performed at the first attempt but was complicated by an extreme bradycardia treated with a single atropine IV bolus of 0.5 mg. We also noticed a transient desaturation as low as SpO_2_ 15% raised to 80% after 5 min of mechanical ventilation. The first end‐tidal capnometry (ETCO_2_) showed an extreme hypercapnia at 154 mmHg (normal range 30–40 mmHg). To improve ventilation, curarization was maintained with an IV bolus of rocuronium 100 mg.

**FIGURE 1:**
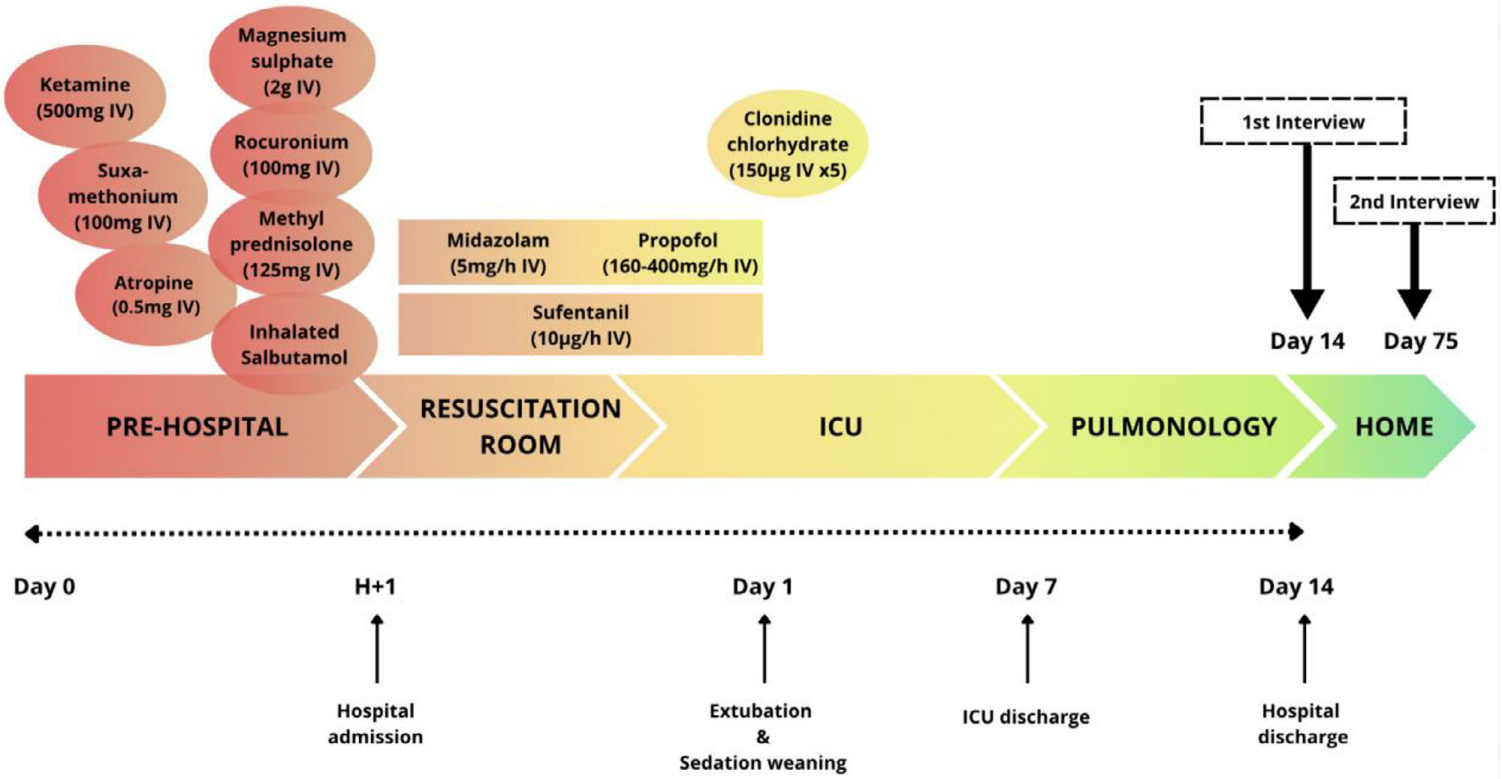
Visual overview of the patient's clinical course and drug administration throughout her hospital stay. The figure illustrates the temporal distribution of sedative, analgesic, and other treatments with psychotropic properties administered from the acute phase through resuscitation and ICU stay, along with the sequence of care transitions. Sedation weaning and extubation occurred on day 1. The first follow‐up interview was conducted on the day of hospital discharge, and the second 2 months after. ICU: intensive care unit; IV: intravenous.

She was admitted to the university hospital's resuscitation room one hour later with an SpO_2_ of 100% and stable hemodynamic parameters. The first blood gases analyses showed an uncompensated respiratory acidosis with a pH of 6.99 (normal range 7.35–7.45), a partial arterial pressure of carbon dioxide (PaCO_2_) of 147 mmHg (normal range 35–48), and partial arterial pressure of oxygen (PaO_2_) of 223 mmHg. Base excess was normal (0.4 mmol/L) as was lactatemia (60 mg/L). Sedation was then maintained with midazolam, sufentanyl, and propofol, and she was extubated upon awakening 24 h after admission to the resuscitation room in the ICU. She remained in the ICU for 7 days before being transferred to the pulmonology department, where she spent another 7 days prior to hospital discharge (see Figure 1).

### 2.2 Procedure and Materials

The patient underwent two semi‐structured interviews: at the hospital on the day of her hospital discharge (i.e., 14 days post‐emergency admission) and at home 2 months post‐emergency admission.

The first interview included questions related to socio‐demographic characteristics (i.e., gender, age at interview, nationality, highest educational level attained) and an explicit free recall task during which the patient was asked to freely recall and describe everything she experienced and could remember about her emergency care. Standardized scales were also administered (see Supporting Information for details on questionnaires). The patient underwent the Galveston Orientation and Amnesia Test (GOAT) (Levin et al. 1979) to confirm adequate orientation to time and space and the Near‐Death‐Experience Content (NDE‐C) scale (Martial, Simon, et al. 2020), permitting the detection of a potential NDE. Additionally, her husband attended the initial interview and provided supplementary information about her state after the event.

In the second interview, the NDE‐C scale was administered again, along with the Montreal Cognitive Assessment (MoCA) (Nasreddine et al. 2005), a screening tool for mild cognitive impairment. The EDS (Sleight et al. 2023) was used to assess the extent to which individuals experienced a loss of their usual sense of self or boundaries between themselves and the external world. We also used the Dissociative experiences scale II (DES‐II) (Carlston and Putnam 1993) to assess the dissociation propensity. Additionally, the Memory Characteristics Questionnaire (MCQ) was used to assess the qualitative characteristics of memories (D'Argembeau and Van der Linden 2008). Finally, the patient was asked to freely recall the experience and to complete a series of open‐ended and multiple‐choice questions related to her NDE adapted from Martial, Fritz, et al. 2024. These specifically designed questions investigate the impression of reality of NDE as well as the potential impact of the experience on her life, well‐being, self‐acceptance, relationship with others, opinion on religion and love, mental awareness, and the meaningfulness of the experience.

## 3 Results

### 3.1 First Interview (day 14)

#### 3.1.1 Free Recall

The patient started by describing her subjective experience, explaining how she found herself “above,” in a space filled with white light and people dressed in white. She reported that these figures seemed to be family members, one of whom held her back, conveying that it was not yet her time to go. She further described an OBE where she could see herself from above lying in a coffin. The patient expressed how profoundly positive this experience had been for her, sharing that, ever since, she sings daily to express the love she felt during the event. Her husband corroborated this, noting that she has regained a sense of joy in life that had been absent for several years. Finally, she added that she now occasionally senses a presence, as if someone is watching over her.

#### 3.1.2 Questionnaires

Table 1 presents the scores of the standardized questionnaires. The patient exhibited good orientation in time and space, as indicated by her score on the GOAT (see also Table S1 for details on item scores). The NDE‐C scale quantified the dissociated state of consciousness she described during the explicit free recall task. With a score of 34/80, the patient's experience met the scale's threshold for identifying an NDE, namely ≥ 27/80 (see Table 1).

**TABLE 1:** Questionnaire scores for the two follow‐up interviews.

Scales	Threshold/cut‐off score	Items	Scores	
			First interview	Second interview
**Near‐Death Experience Content scale**	≥ 27/80 0 = not at all; none 1 = slightly 2 = moderately 3 = strongly; equivalent in degree to any other strong experience lived so far 4 = extremely; more than any other time in my life and stronger than 3	Time perception	**4**	**0**
		Speeded thoughts	0	0
		Voice	0	0
		Understanding	**0**	**4**
		Peacefulness/well‐being	4	4
		Harmony/unity	**4**	**0**
		Bright light	4	4
		Unusual sensation	**2**	**4**
		Extrasensory perception	0	0
		Precognition	0	0
		Out‐of‐body experience	0	0
		Leaving the earthly world	**2**	**0**
		Life review	0	0
		Encounter	**2**	**4**
		Non‐existence/void/fear	0	0
		Border/point of no return	**4**	**0**
		Come back	4	4
		Dying	0	0
		Gateway	**0**	**4**
		Ineffability	4	4
		**Total score**	**34**	**32**
**Ego Dissolution scale**	No cut‐off score	Ego‐loss subscore	—	50
		Unity subscore	—	75
		**Total score**	—	**60**
**Dissociative Experience scale**	> 15/100 = pathological dissociation	DES‐T score	—	0
	> 25/100 = tendency to dissociation traits	**Total score**	—	**0.36**
**Galveston Orientation and Amnesia Test**	76 à 100 = normal < 66 = impaired	**Total score**	**89**	—
**Montreal Cognitive Assessment**	26‐30 = normal 18–25 = mild cognitive impairment 10–17 = moderate cognitive impairment < 10 = severe cognitive impairment	**Total score**	—	**14**
**Memory Characteristics Questionnaire**	1 = not at all 7 = completely	Vividness	—	7
	1 = none 7 = a lot	Visual details	—	7
	1 = none 7 = a lot	Sensory details	—	1
	1 = not at all 7 = very clearly	Place details	—	1
	1 = not at all 7 = very clearly	Time details	—	1
	1 = not at all 7 = completely	Narrative coherence	—	7
	1 = not at all 7 = a lot	Verbal component	—	1
	1 = not at all 7 = completely	Emotional intensity	—	7
	1 = 100% imaginary 7 = 100% real	Belief in accuracy	—	7
	1 = not at all 7 = very clearly	Actions recall	—	1
	1 = not at all 7 = very clearly	Verbal recall	—	1
	1 = not at all 7 = very clearly	Thoughts recall	—	1
	1 = totally observer 7 = totally actor	Observer perspective	—	7
	1 = very negative 7 = very positive	Emotional valence	—	7
	1 = not at all important 7 = very important	Significance	—	7
	1 = not at all 7 = very often	Sharing	—	3

*Note*: Grey‐shaded cells indicate total scores for each questionnaire. In the NDE‐C scale, bold values represent the change in score between the first and second interview.

### 3.2 Interview (day 75)

#### 3.2.1 Free Recall

The patient expressed similar subjective features and emotions as described in the first interview. She also reiterated her description of the frequent sense of presence she experiences; the entity is consistently perceived as the same. However, unlike in the first interview, she did not mention the OBE, neither explicitly nor in response to probing.

#### 3.2.2 Questionnaires

As shown in Table 1, the patient obtained a score of 0.36 on the DES, indicating a very minimal level of dissociative symptoms. She reported relatively high scores on the EDS (see Tables S3 and S4 for detailed item scores). Although the NDE‐C total score remained relatively stable, showing only a two‐point decrease (from 34 to 32), eight prototypical features shifted across the two interviews, either by appearing or disappearing, or by showing a change in intensity of scoring. For example, the item “Gateway,” which captures the sensation of crossing through a transition or entry point (e.g., tunnel, door), was absent for the first interview, while she scored 4 for the second one. On the MCQ, using a Likert scale from 1 to 7, half of the items were rated at the maximum score, including vividness, visual details, narrative coherence, emotional intensity, emotional valence, belief in accuracy, and significance. The other half were all rated at the minimum score, except for the sharing item, which was rated moderately with a score of 3. Moreover, the patient's responses to multiple‐choice questions indicate a significant enhancement in the evaluation of love, self‐acceptance, and empathy as compared to her state prior to the acute event (see Table 2). Additionally, we note a subjective indication of an expanded consciousness, as she perceives a deeper understanding of life's meaning and what may follow thereafter. Finally, the patient's cognitive abilities, as assessed by the MoCA, indicate moderate cognitive impairment (see Table S4 for detailed item scores).

**TABLE 2:** Responses to multiple‐choice questions related to NDE (second interview).

Questions	Response possibilities	Response of the patient
How often do you think back about what you experienced?	1: Never … 7: All the time	4
To what extent did the experience hold personal significance for you?	0: Not more than usual … 5: The most significant experience of my life	5
Did the experience have a positive impact on your life?	0: No positive impact … 5: Very significant positive impact	5
Did the experience have a positive effect on your well‐being?	0: No positive impact … 5: Very significant positive impact	5
Did the experience have a significant effect on your self‐acceptance?	0: Highly decreased self‐acceptance … 5: Highly increased self‐acceptance	5
Did the experience have a significant effect on your relationship with others?	0: No effect … 5: I'm more oriented toward others	0
Did the experience have a negative impact on your life?	0: No negative impact … 5: Very significant negative impact	0
Did the experience have a negative impact on your well‐being?	0: No negative impact … 5: Very significant negative impact	0
Since this experience, would you say that you feel alienated from the world, as if you no longer belong?	0: Not at all … 5: Completely	0
Since this experience, would you say that you feel more anxious?	0: Not at all … 5: Completely	0
Since this experience, would you say that you have more insomnia or nightmares?	0: Not at all … 5: Completely	0
Since this experience, would you say that you eat less or less healthy?	0: Not at all … 5: Completely	0
Did the experience allow you to expand your mental awareness?	0: Not at all … 5: Fully more expanded mental awareness	5
While living the experience, did you consider it “real” (i.e., different from a dream or a hallucination)?	−3%–100% dream/hallucination … 0: Usual reality … 3:Much more real than usual	3
Now, do you consider that the experience is “real” (i.e., different from a dream or a hallucination)?	−3%–100% dream/hallucination … 0: Usual reality … 3: Much more real than usual	3
According to you, does this experience provide evidence for the existence of an afterlife?	1: Yes 2: No 3: I do not know	1
Had you ever experienced this type of event before your hospitalization?	1: Yes 2: No/I do not remember	2
Since your stay in the ICU, have your beliefs/opinions changed regarding death?	1: I am much more afraid of death 2: I am more afraid of death 3: No change 4: I am less afraid of death 5: I am much less afraid of death	3
Since your stay in the ICU, have your beliefs/opinions changed regarding religion?	1: Much less religious beliefs 2: Less religious beliefs 3: No change 4: More religious beliefs 5: Much more religious beliefs	5
Since your stay in the ICU, have your beliefs/opinions changed regarding love?	1: Significantly reduced sense of love 2: Reduced sense of love 3: No change 4: Increased sense of the importance of love 5: Greatly increased sense of the importance of love	5

## 4 Discussion

This report details a 73‐year‐old patient with chronic COPD who, after severe acute respiratory distress and accidental administration of 500 mg ketamine during intubation, experienced an NDE as reported in a post‐care interview.

### 4.1 Multifactorial Origins of NDEs

While the patient's score on the NDE‐C scale confirms that she had an NDE, it is not possible to disentangle the exact cause(s) from the gamut of possible contributing pharmacological and physiological factors. The patient had hypoxia, hypercapnia, and bradycardia and received a high dose of ketamine, any or all of which could potentially be contributing factors to her NDE (Klemenc‐Ketis et al. 2010; Lempert et al. 1994; Martial, Cassol, et al. 2020). Interestingly, parallels between the effects of ketamine and NDEs were noted as early as 1997 by Jansen (1997a), hypothesizing the existence of a speculative endogenous ketamine‐like neurotoxin that might be released in critical situations. This neurotoxin was suggested to act as an antagonist to NMDA receptors, producing subjective experiences like those induced by ketamine, potentially explaining the phenomenological similarities between the two experiences (Martial et al. 2019). However, no empirical evidence to date supports this hypothesis. Jansen later refined his view, suggesting that ketamine may be better understood as one possible pathway through which NDEs can occur, emphasizing a permissive rather than a strictly causative role (Jansen 1997b). Although this patient's experience was likely shaped by both ketamine and a pre‐existing hypoxic‐hypercapnic state, it remains unknown whether an NDE would have occurred in the absence of ketamine. Given this high dose and the known phenomenological overlap between ketamine‐induced experiences and NDEs, ketamine alone may have been sufficient to induce a number of prototypical features, potentially enhancing or even triggering the experience (Martial et al. 2019). Moreover, and in contrast to previous findings suggesting a higher propensity for dissociation among NDE experiencers (Greyson 2000; Rousseau et al. 2023), this patient scored low on the DES‐II, indicating minimal dissociative tendencies. However, personal beliefs—for example, the fact that the patient described herself as a believer before the acute event—as well as her psychological state and expectations prior to the event were not assessed, and we cannot exclude that such factors may also have contributed to the NDE. Overall, this supports the hypothesis that the phenomenology observed in this case was potentially more strongly driven by the physiological state of the patient as well as the pharmacological effects of ketamine than by cognitive predispositions. In fact, ketamine disrupts the brain's excitation and inhibition balance by blocking NMDA receptors, subsequently reducing GABA release from interneurons and increasing the activity in excitatory pyramidal neurons (Martial et al. 2025). This pharmacological cascade can induce perceptual changes and disembodiment including OBE and feelings of awe, positivity, and interconnectedness (Martial et al. 2019). These features are commonly reported during both ketamine experiences and NDEs, as also described by this patient. This is particularly important to consider, as many patients receive psychoactive drugs during critical care without being aware of it (e.g., opioids such as fentanyl, benzodiazepines such as midazolam, or anesthetics such as propofol and dexmedetomidine) and may later report NDEs without recognizing the potential pharmacological contribution, possibly leading to under‐reporting of drug‐induced experiences in the literature (Ferguson et al. 2022; Klein et al. 2018; Marchaisseau et al. 2008; Radek et al. 2018). While ketamine‐induced hyperexcitability is one possible cause, neuronal hyperactivity can also occur under hypoxic conditions, where a glutamate surge could activate AMPA receptors, potentially overriding inhibitory control and leading to neuronal hyperexcitability (Höflich et al. 2017). In the case of our patient, the fact that this high dose of ketamine was administered concurrently with severe hypoxia likely enhanced the effect by blocking NMDA receptors. This combined disruption of inhibitory control and excessive excitation may have contributed to increased neuronal hyperexcitability, possibly explaining the rich phenomenology and experience of ego‐dissolution.

### 4.2 Memory Stability and Recall Variability

While identifying the precipitating factors for this NDE presents a challenge, it is also important to consider the vividness and persistence of the patient's memory. Given the life‐threatening nature of the situation in which she experienced the NDE, her responses on the MCQ were moderate, indicating a relatively vivid and detailed recollection, with some emotional and visual hallucinatory intensity, yet not reaching the levels typically observed in NDEs (Martial et al. 2017; Thonnard et al. 2013). Moreover, although her total NDE‐C scores remained relatively stable between the two interviews (32 and 34, respectively), this apparent global stability contrasts with substantial variations in the specific content of her experience. For instance, NDE‐C features such as “a sudden understanding of oneself, others, or the universe” and “entering a gateway” emerged only in the second interview, while other dimensions such as the sense of harmony or unity were no longer reported. In particular, during the first interview, the patient provided a detailed subjective description of an OBE in which she perceived herself to be above her body, which appeared to her to be lying in a coffin, with no real‐life‐based corroboration. Interestingly, this experience was not reflected in her response to the corresponding NDE‐C item (“You felt separated from your body”). In the second interview, the OBE was no longer reported, either in the free recall or on the NDE‐C scale, suggesting selective memory retention. This observation first raises questions about both the stability of NDE memories and the suitability of the total NDE‐C score as an indicator to reflect it, particularly in the early post‐acute period. While the limited retrospective literature suggests that NDEs remain stable both over short‐term intervals (Greyson 1983) and over periods of up to 20 years in NDE experiencers (Greyson 2007), in both cases the first assessment occurred on average 17–19 years after the NDE. These findings therefore evaluate stability in memories that had already undergone long‐term consolidation. In contrast, we report here the first evidence of content‐related changes as early as 2 months post‐event, based on assessment starting immediately after the acute episode. This difference in timing may explain the discrepancy, as early memories may be more susceptible to change before long‐term consolidation occurs. This highlights the need to examine item‐level changes and to conduct prospective studies, as narrative content may evolve over time, especially in early post‐acute assessments (Schacter et al. 2011). Second, the moderate cognitive impairment identified by the MoCA may suggest potential limitations in her ability to fully understand instructions and/or engage in metacognitive reasoning during self‐assessment, particularly in an acute setting. Cognitive factors such as attention, abstraction, or executive functioning, all assessed and affected in the MoCA, may influence how she recalls and translates the experience into responses to simple or complex questionnaire items. Such cognitive limitations may distort the recall of complex or emotionally charged content. To ensure there was no major underlying brain pathology, we reviewed her neuroimaging history: an MRI performed in 2021 showed only mild chronic microvascular leukoencephalopathy (Fazekas grade 1) with a right periventricular frontal microbleed. CT scans from 2022 and 2024 revealed no clear signs of progression, though direct comparison was limited by differing imaging modalities. Based on these findings, we do not suspect any underlying neurodegenerative disorder.

Despite these potential cognitive limitations, the patient reported significant changes in her daily life following the NDE. At the first interview, 2 weeks after the event, both she and her husband reported that she had begun to display behaviors typically associated with joy, such as singing daily, something she had not done for years prior to the event. In addition, they both described a noticeable improvement in her mood, describing her as more cheerful and smiling more often. These positive changes persisted into the second interview, during which the patient also described becoming more sensitive, with an increased sense of empathy, and expressed a heightened belief in love and its importance. These changes align with commonly reported aftereffects of NDEs (Greyson 2022; Groth‐Marnat and Summers 1998).

### 4.3 Clinical Implications

Although NDEs are known to occur in up to 23% of patients in acute medical contexts (Schwaninger et al. 2002), they remain under‐recognized and rarely documented in routine clinical care. Patients may leave hospital with intense subjective experiences, whether perceived as positive or distressing, that can be difficult to integrate into everyday life. These experiences can hold profound personal significance and pose challenges for psychological integration, especially when they induce substantial changes in beliefs or behaviors—affecting not only the individual but also their relatives, who may suddenly perceive significant changes in the person. This case highlights the importance of raising clinicians' awareness of the clinical reality of NDEs. Easy‐to‐use, fast tools such as the NDE‐C scale could help clinicians to identify patients who have had such experiences, giving them the opportunity to talk about it, receive appropriate support if necessary, and develop a better understanding of their experience. Beyond improving individual care, systematic screening could facilitate research by enabling the prospective collection of standardized data. This would enable specific physiological conditions or administered medications to be associated with the occurrence of NDEs, thereby improving our understanding of the contributing factors and supporting future prospective studies. Although such existential shifts are less documented with ketamine per se, the substance has been linked to lasting improvements in mood and behavior, especially in depression and addiction treatment (Bahji et al. 2021; Dakwar et al. 2018).

### 4.4 Limitations

In this case, it is not possible to distinguish the pharmacological effects of ketamine from the concurrent neurophysiological changes related to the patient's acute medical condition. Furthermore, the patient was enrolled during the pilot phase of a larger study, before the full research protocol had been finalized. Certain procedures, such as repeating standardized questionnaires at follow‐up, had not yet been fully implemented. These tools could have helped to document changes in the patient's subjective narrative and symptoms over time, for example, after 6 months or 1 year. While no generalization can be drawn from a single case, this case highlights the need for prospective studies with structured follow‐up to better characterize the phenomenological features of such experiences and explore their potential neurophysiological and pharmacological contributing factors.

### 4.5 Conclusion

We report the case of a hemodynamically unstable patient with a hypoxic exacerbation of COPD and severe hypercapnia who received a higher‐than‐usual dose of ketamine and subsequently reported an NDE with lasting positive changes. This case illustrates how NDE can emerge from the interaction between pharmacological agents and the patient's neurophysiological state. It highlights the need to consider not only the drug itself but also the broader physiological context in which it is administered. The vivid memory and the positive personal changes reported by the patient underscore the profound and lasting impact such experiences can have but also the potential of ketamine to facilitate positive experiences in highly stressful and life‐threatening contexts. However, despite apparent stability in overall NDE‐C scores over time, prospective item‐by‐item analysis revealed significant variations, emphasizing the importance of fine‐grained longitudinal approaches for capturing the dynamics of NDE recall. Prospective studies will be essential to improve our understanding of NDEs and patient care in (post‐)critical situations.

## Author Contributions

Pauline Fritz, Anaïs Pichelin, and Charlotte Martial conceptualized the review, wrote the article, and edited the manuscript before submission. All authors contributed substantially to discussion of the content and reviewed and edited the manuscript before submission. All authors approved the version to be published.

## Ethics Statement

This study was approved by the hospital‐university ethics committee of Liège (approval no. 2022–389) in March 2023

## Consent

Written informed consent was obtained from the patient on the 17/07/2023. Please note that non‐essential identifying details have been omitted to ensure confidentiality.

## Conflicts of Interest

The authors declare no conflicts of interest.

## Peer Review

The peer review history for this article is available at https://publons.com/publon/10.1002/brb3.70939.

## Supporting information




**Supplementary Materials**: brb370939‐sup‐0001‐SuppMatt.docx

## Data Availability

The data that support the findings of this study are available from the corresponding author upon request and after meeting the condition established by the local ethical committee (i.e., data sharing agreement).
