# Supplementary material for: Near‐Death Experience During Emergency Ketamine Use: A Case Report
Source: Brain Behav. 2025 Oct 24;15(10):e70939. doi: 10.1002/brb3.70939 (PMC12551671; doi:10.1002/brb3.70939)
Supplement: Supplementary file 1 — Supplementary Materials: brb370939‐sup‐0001‐SuppMatt.docx [file BRB3-15-e70939-s001.docx]

**Supplemental Materials**

Questionnaires

* The Galveston Orientation and Amnesia Test (GOAT; Levin et al., 1979) is a structured clinical interview developed to assess cognitive recovery following a closed head injury. The test comprises 15 questions designed to measure orientation to person, place, and time, as well as memory for events preceding and following the injury. Each response contributes to a total score ranging from 0 to 100, with higher scores indicating better orientation and memory functioning. Scores are interpreted as follows: scores ≤ 66 reflect impaired orientation, scores between 66 and 75 indicate borderline performance, and scores between 76 and 100 are considered within the normal range. The GOAT is typically administered daily during early recovery, and a score above 76 for three consecutive days is commonly used to determine emergence from post-traumatic amnesia.

* The Near-Death Experience Content (NDE-C) scale (Martial et al., 2020) is a 20-item self-report questionnaire designed to systematically assess the phenomenological content of near-death experiences (NDEs). Each item is rated on a 5-point Likert scale: 0 corresponds to “not at all; none,” 1 to “slightly,” 2 to “moderately,” 3 to “strongly; equivalent in degree to any other strong experience,” and 4 to “extremely; more than any other time in my life and stronger than 3.” Total scores range from 0 to 80, with higher scores reflecting richer and more intense NDE content. A total score of 27 or above is used as a threshold for identifying the occurrence of a potential NDE. The scale encompasses five subscales reflecting key experiential dimensions: Beyond the Usual, Harmony, Insight, Border, and Gateway.

* The Ego Dissolution Scale (EDS; Sleight et al., 2023) is a 10-item self-report questionnaire developed to assess the subjective experience of ego dissolution, a phenomenon often reported during altered states of consciousness. The patient was asked to indicate the extent to which each statement applies to her experience, using a continuous scale from 0% to 100%, where 0% represents “not more than usual” (i.e., a normal state of consciousness) and 100% corresponds to “yes, completely.” The scale encompasses two dimensions: Ego-Loss, reflecting the subjective experience of self-loss (mean of items 1–6), and Unity, capturing positive experiences of oneness and connection typically linked to mystical states (mean of items 7–10). An overall total score was calculated as the average of all items. The usage of the EDS is justified by the fact that ketamine is commonly classified as a dissociative anesthetic, yet its unique phenomenological profile—sharing considerable overlap with classical psychedelics—complicates its categorization. Ketamine can induce dissociative effects from the environment, physical body, or sense of self, which are broadly captured by the EDS. It can also induce changes in the narrative self, which may manifest as ego dissolution and a profound sense of interconnectedness—characteristics more commonly associated with classical psychedelics and captured by the EDS.

* The Montreal Cognitive Assessment (MoCA; Nasreddine et al., 2005) is a 30-point cognitive screening tool designed to detect mild cognitive impairment. Administered in approximately 10 minutes, it assesses multiple domains: short-term memory, visuospatial abilities, executive functions, attention and working memory, language, and orientation to time and place. The total score ranges from 0 to 30, with higher scores indicating better cognitive functioning. Interpretation thresholds are as follows: 26–30 = Normal, 18–25 = Mild cognitive impairment, 10–17 = Moderate cognitive impairment, and <10 = Severe cognitive impairment.

* The French version (D’Argembeau and Van der Linden, 2008) of the Memory Characteristics Questionnaire (MCQ; Johnson et al., 1988) is built to analyze phenomenological characteristics of real and imagined memories. This questionnaire encompasses 16 rating scales assessing feeling of re-experiencing, visual details, other sensory details, location, time, coherence, verbal component, emotion while remembering, belief that the event is real, one’s own actions, words and thoughts, visual perspective, emotional valence, personal importance, and reactivation frequency. A MCQ total score was derived summing all the 16 items (each on a 1–7 point Likert scale) and referred to as the amount of memory characteristics (i.e., higher total scores reflect greater amount of memory characteristics).

* The French version (back-translated method) of the 28-item Dissociative experiences scale II (DES-II; Carlston and Putnam, 1993) permits to assess frequency and types of both pathological and non-pathological dissociative experiences. Each item describes a specific daily life experience, reflecting different forms of dissociative symptoms (depersonalization and derealization, absorption/imaginative involvement, dissociative amnesia). The patient was asked to report the percentage of the time she had the experience described, on a scale ranging from 0% (never) to 100% (always). Each item of the scale is rated from 0 to 100 and the final score is obtained by calculating the average of all item ratings. A score ≥25 indicates a tendency to dissociation trait. A DES-T score can also be calculated to identify pathological dissociation (cut-off score of 15) (Waller et al., 1996), which corresponds to the average of eight items (items 3, 5, 7, 8, 12, 13, 22, and 27).

**Supplemental Table 1. Scores of the Galveston Orientation and Amnesia Test** **(first interview)**

| **Question** | **Error Score** | **Notes** |
| --- | --- | --- |
| What is your name? | 0/2 | Must give both first name and surname. |
| When were you born? | 0/4 | Must give day, month, and year. |
| Where do you live? | 0/4 | Town is sufficient. |
| Where are you now? |  |  |
| (a) City | 0/5 | Must give actual town. |
| (b) Building | 0/5 | Usually in hospital or rehab center. |
| When were you admitted to this hospital? | 5/5 | Date. |
| How did you get here? | 0/5 | Mode of transport. |
| What is the first event you can remember after the injury? | 0/5 | Any plausible event is sufficient |
| Can you give some detail? | 0/5 | Must give relevant detail. |
| Can you describe the last event you can recall before the accident? | 0/5 | Any plausible event is sufficient |
| What time is it now? | 4/5 | 1 for each half-hour error, etc. |
| What day of the week is it? | 0/3 | 1 for each day error, etc. |
| What day of the month is it? (i.e. the date) | 2/5 | 1 for each day error, etc. |
| What is the month? | 0/15 | 5 for each month error, etc. |
| What is the year? | 0/30 | 10 for each year error. |
| **Total Error** | **11** |  |
| **100 - Total Error** | **89** | Can be a negative number. |

**Supplemental Table 2.** S**cores of the Montreal Cognitive Assessment scale (second interview)**

| **Cognitive domains** | | **Score** |
| --- | --- | --- |
| Visuospatial / Executive | *Alternating Trail Making* | 0/1 |
|  | *Cube* | 0/1 |
|  | *Clock* | 0/3 |
|  | *Total* | 0/5 |
| Naming | | 3/3 |
| Memory | | No points |
| Attention | | 0/2 |
| Language | *Sentence repetition* | 1/2 |
|  | *Verbal fluency* | 0/1 |
|  | *Total* | 1/3 |
| Abstraction | | 0/2 |
| Delayed recall | | 4/5 |
| Orientation | | 3/6 |
| **Total** | | **14/30** |

**Supplemental Table 3. Scores of the Ego Dissolution Scale (second interview)**

| **Items** | **Score (out of 100%, in increments of 10** |
| --- | --- |
| 1. My “self” or ego dissolves into nothingness. | 100 |
| 2. My “self” disappears and no “me” or “I” is present any longer. | 100 |
| 3. I feel I do not exist. | 100 |
| 4. I experience being out of my body. | 0 |
| 5. I experience a disintegration of my “self” or ego. | 0 |
| 6. My sense of self moved from one part of my body to another (i.e., from behind my eyes to my heart). | 0 |
| 7. I feel at one with the universe. | 0 |
| 8. I feel one with everything around me. | 100 |
| 9. I feel a sense of union with others. | 100 |
| 10. I feel I merge with others/the world. | 100 |
| **Total Score** | **60** |

**Supplemental Table 4. Scores of the Dissociative experiences scale-II (second interview)**

| **Items** | **Score (out of 100%, in increments of 10)** |
| --- | --- |
| Some people have the experience of driving or riding in a car or bus or subway and suddenly realizing that they don’t remember what has happened during all or part of the trip. | 0 |
| Some people find that sometimes they are listening to someone talk and they suddenly realize that they did not hear part or all of what was said. | 10 |
| Some people have the experience of finding themselves in a place and have no idea how they got there. | 0 |
| Some people have the experience of finding themselves dressed in clothes that they don’t remember putting on. | 0 |
| Some people have the experience of finding new things among their belongings that they do not remember buying. | 0 |
| Some people sometimes find that they are approached by people that they do not know, who call them by another name or insist that they have met them before. | 0 |
| Some people sometimes have the experience of feeling as though they are standing next to themselves or watching themselves do something and they actually see themselves as if they were looking at another person. | 0 |
| Some people are told that they sometimes do not recognize friends of family members. | 0 |
| Some people find that they have no memory for some important events in their lives (for example, a wedding or graduation). | 0 |
| Some people have the experience of being accused of lying when they do not think that they have lied. | 0 |
| Some people have the experience of looking in a mirror and not recognizing themselves. | 0 |
| Some people have the experience of feeling that other people, objects, and the world around them are not real. | 0 |
| Some people have the experience of feeling that their body does not seem to belong to them. | 0 |
| Some people have the experience of sometimes remembering a past event so vividly that they feel as if they were reliving that event. | 0 |
| Some people have the experience of not being sure whether things that they remember happening really did happen or whether they just dreamed them. | 0 |
| Some people have the experience of being in a familiar place but finding it strange and unfamiliar. | 0 |
| Some people find that when they are watching television or a movie they become so absorbed in the story that they are unaware of other events happening around them. | 0 |
| Some people find that they become so involved in a fantasy or daydream that it feels as though it were really happening to them. | 0 |
| Some people find that they sometimes are able to ignore pain. | 0 |
| Some people find that they sometimes sit staring off into space, thinking of nothing, and are not aware of the passage of time. | 0 |
| Some people sometimes find that when they are alone they talk out loud to themselves. | 0 |
| Some people find that in one situation they may act so differently compared with another situation that they feel almost as if they were two different people. | 0 |
| Some people sometimes find that in certain situations they are able to do things with amazing ease and spontaneity that would usually be difficult for them (for example, sports, work, social situations, etc.). | 0 |
| Some people sometimes find that they cannot remember whether they have done something or have just thought about doing that thing (for example, not knowing whether they have just mailed a letter or have just thought about mailing it). | 0 |
| Some people find evidence that they have done things that they do not remember doing. | 0 |
| Some people sometimes find writings, drawings, or notes among their belongings that they must have done but cannot remember doing. | 0 |
| Some people sometimes find that they hear voices inside their head that tell them to do things or comment on things that they are doing. | 0 |
| Some people sometimes feel as if they are looking at the world through a fog, so that people and objects appear far away or unclear. | 0 |
| **Total Score** | **0.36** |

**References**

Carlson, E. B., Putnam, F. W. (1993). An update on the Dissociative Experiences Scale. *Dissociation: Progress in the Dissociative Disorders, 6*(1), 16–27.

D'Argembeau, A., Van der Linden, M. (2008). Remembering pride and shame: self-enhancement and the phenomenology of autobiographical memory. *Memory, 16*(5), 538-47. doi: 10.1080/09658210802010463

Johnson, M.K., Foley, M.A., Suengas, A.G., Raye, C.L. (1988). Phenomenal characteristics of memories for perceived and imagined autobiographical events. *J Exp Psychol Gen, 117*(4), 371-6. [https://doi.org/10.1037/0096-3445.117.4.371](https://psycnet.apa.org/doi/10.1037/0096-3445.117.4.371)

Levin, H.S., O'Donnell, V.M., Grossman, R.G. (1979). The Galveston Orientation and Amnesia Test. A practical scale to assess cognition after head injury. *J Nerv Ment Dis*, *167*(11), 675-84. doi: 10.1097/00005053-197911000-00004

Martial, C., Simon, J., Puttaert, N., Gosseries, O., Charland-Verville, V., Nyssen, A.-S., … Cassol, H. (2020). The Near-Death Experience Content (NDE-C) scale: Development and psychometric validation. *Consciousness and Cognition*, *86*, 103049. <https://doi.org/10.1016/j.concog.2020.103049>

Nasreddine, Z.S., Phillips, N.A., Bédirian, V., Charbonneau, S., Whitehead, V., Collin, I., Cummings, J.L., Chertkow, H. (2005). The Montreal Cognitive Assessment, MoCA: a brief screening tool for mild cognitive impairment. *J Am Geriatr Soc, 53*(4), 695-9. doi: 10.1111/j.1532-5415.2005.53221.x

Sleight, F.G., Lynn, S.J., Mattson, R.E., McDonald, C.W. (2023). A novel ego dissolution scale: A construct validation study. *Conscious Cogn, 109,* 103474. doi: 10.1016/j.concog.2023.103474

Waller, N.G., Putnam, F.W., Carlson, E.B. (1996). Types of dissociation and dissociative types: A taxometric analysis of dissociative experiences. *Psychological Methods, 1*(3), 300-321. [https://doi.org/10.1037/1082-989X.1.3.300](https://psycnet.apa.org/doi/10.1037/1082-989X.1.3.300)
